# Exploring Young Adults' Reluctance to Engage With Psychiatric Hospitals in Erbil, Iraq: Identifying Barriers to Psychiatric Care

**DOI:** 10.7759/cureus.62164

**Published:** 2024-06-11

**Authors:** Sangar M Ahmed, Abdulmalik F Saber, Ahmed A Naif, Abdulqader H Hamad, Sirwan K Ahmed, Ammar Y Abdullah, Karzan Qurbani, Safin Hussein

**Affiliations:** 1 Department of Public Health, College of Health Sciences, Hawler Medical University, Erbil, IRQ; 2 Department of Nursing, Faculty of Nursing, Tishk International University, Erbil, IRQ; 3 Department of Psychiatric and Mental Health Nursing, College of Nursing, Hawler Medical University, Erbil, IRQ; 4 College of Nursing, University of Raparin, Sulaymaniyah, IRQ; 5 Department of Biology, University of Raparin, Sulaymaniyah, IRQ

**Keywords:** psychiatric aides, barriers to care, psychiatric hospitals, reluctance, young adults

## Abstract

Background and aim: In Erbil, Iraq, the reluctance of young adults to engage with psychiatric services is influenced by a complex array of barriers, including stigma-related, attitudinal, and instrumental factors that hinder effective mental healthcare access. This study aimed to identify these specific barriers to accessing psychiatric care among young adults in Erbil.

Materials and methods: The study utilized a cross-sectional online survey conducted between April 5th and May 1st, 2024. Data collection was carried out through purposive sampling and involved a comprehensive questionnaire. Electronic informed consent was obtained from all participants before they started the survey, which collected demographic data and utilized the Barriers to Access to Care Evaluation (BACE v3) tool. Statistical analysis was conducted using SPSS version 27 (IBM Corp., Armonk, NY). Descriptive statistics (frequency and percentage) were used for categorical data, while the mean and standard deviation characterized continuous variables. Chi-square tests, including Fisher’s exact test and odds ratio (OR), were used to analyze categorical data, with a significance level set at p < 0.05.

Results: A total of 407 participants were enrolled in the study. The study highlighted several barriers to mental health care. Stigma-related barriers were significant, with participants fearing being seen as weak (mean score = 2.14, SD = 0.96) and concerns about being labeled "crazy" (mean score = 1.80, SD = 1.19). Regarding attitudinal barriers, there was a notable preference for dealing with issues independently (mean score = 2.04, SD = 0.98) and a tendency toward resolving problems without professional help (mean score = 1.88, SD = 0.98). Additionally, instrumental barriers were identified, including the rare availability of culturally diverse mental health professionals (mean score = 1.78, SD = 1.09) and practical difficulties such as arranging transportation to appointments (mean score = 0.61, SD = 0.87).

Conclusion: The study demonstrated that young adults in Erbil face significant stigma and attitudinal and instrumental barriers to accessing psychiatric care. In response to these findings, it is recommended for the government to prioritize mental health awareness, actively destigmatize mental health issues, and improve service accessibility to foster a supportive care environment. Additionally, mental health professionals and educational institutions should collaborate to provide targeted support programs and resources for young adults.

## Introduction

Mental health disorders, including psychiatric conditions, are a substantial worldwide health issue. According to the World Health Organization (WHO), there are roughly 450 million individuals globally who have mental or neurological problems [1,2]. In Iraq, the burden of mental health issues is substantial. A study estimated that approximately 18.5% of the population suffers from some form of mental disorder [3]. Young adults, typically defined as individuals between the ages of 18 and 35 years, are particularly vulnerable to developing psychiatric conditions such as depression, anxiety, and substance abuse disorders [4]. Because of this vulnerability, it is crucial to provide effective mental health services that cater to their unique needs.

The city of Erbil, located in the Kurdistan region of Iraq, is not exempt from this mental health crisis. Despite the availability of psychiatric hospitals and mental health services in the region, young adults in Erbil seem reluctant to seek help from these facilities. Several factors contribute to this reluctance, including cultural stigma, lack of awareness, and accessibility barriers [5,6]. This situation is particularly concerning because about 75% of mental health conditions emerge by the age of 25 [7], emphasizing the critical need for early intervention and treatment. Failing to address these barriers can lead to delayed psychiatric care, which can worsen mental health conditions and have adverse consequences for individuals, their families, and society as a whole.

The reluctance of young adults in Erbil to engage with psychiatric hospitals could be due to various sociocultural factors. Cultural beliefs, stigma, and misconceptions surrounding mental health significantly influence attitudes and decision-making [8]. Additionally, the lack of mental health literacy and awareness about the availability and benefits of psychiatric services could contribute to this reluctance [9]. Negative perceptions about the quality of care or cultural competency may also deter young people from seeking psychiatric treatment [10]. Practical barriers can also hinder young adults from seeking psychiatric care, such as limited accessibility, transportation challenges, and financial constraints [6]. For example, geographical distances, inadequate public transportation systems, and treatment costs can create significant obstacles, especially for individuals from lower socioeconomic backgrounds or those living in remote areas [11]. Personal factors also play a role in preventing young adults from seeking psychiatric care. They may experience fear, shame, and a desire for autonomy and independence, which can contribute to a reluctance to acknowledge mental health struggles and seek professional help [12]. Furthermore, a lack of trust in the healthcare system or concerns about confidentiality may discourage young adults from disclosing sensitive information about their mental health [13].

There are also systemic issues within the healthcare infrastructure that exacerbate the challenges faced by young adults in accessing psychiatric services. Inadequate mental health resources, including a shortage of qualified mental health professionals, limited inpatient facilities, and insufficient community-based support services, create significant barriers to care [14]. Early intervention at the first manifestation of symptoms can significantly improve the prognosis for various psychiatric disorders. Additionally, a lack of integration between mental health services and primary care settings can contribute to fragmented care and poor coordination, making it difficult for individuals to navigate the system effectively [15].

Despite the growing recognition of mental health issues in Iraq, there is a lack of research specifically exploring the barriers faced by young adults in Erbil when accessing psychiatric care. Without proper information and societal support, young people may internalize cultural stigma and self-stigmatize, viewing seeking mental health care as a personal failure. To effectively tackle this urgent problem, it is crucial to have a thorough comprehension of these barriers. Without such awareness, it becomes difficult to devise precise treatments and solutions. Therefore, this study aimed to investigate the factors contributing to the reluctance of young adults in Erbil to engage with psychiatric hospitals.

## Materials and methods

Study design, setting, period, and sample size

This research was an online cross-sectional study conducted in Erbil, Kurdistan region of Iraq. Data collection occurred from April 5th to May 1st, 2024, using a purposive sampling approach. The sample size was calculated with a 95% confidence interval, an estimated response distribution of 50%, and a 5% margin of error. Using the formula for an infinite population, a Zα/2 value of 1.96 was determined. Although the initial sample size calculation was 385 participants, the sample was expanded to 407 participants due to the availability of additional cases. To ensure broad distribution and participant recruitment, the questionnaire was distributed online via Google Forms. The survey link was shared across platforms such as email lists, WhatsApp, Twitter, and Facebook.

Inclusion/exclusion

The study targeted young adults aged 18 to 25 years who provided electronic informed consent to participate. Exclusion criteria included participants failing to answer one variable, which resulted in their removal from the study.

Study tool

The questionnaire was structured into two sections. The first section gathered sociodemographic data, including age, gender, relationship status, religion, and self-reported current and lifetime mental health issues. The second section employed the Barriers to Access to Care Evaluation (BACE v3) tool to assess barriers to psychiatric care [16]. The BACE v3 consists of 30 statements addressing three main areas: stigma, attitudinal barriers, and instrumental barriers. To ensure accuracy, the questionnaire was translated from English to Kurdish using a forward-backward translation method and reviewed by a psychiatrist. Completing the questionnaire required approximately five minutes.

Pilot study

A pilot study was conducted with 25 participants. This phase, running from February 15 to March 15, assessed the reliability of the questionnaire using Cronbach's alpha [17], which had an overall score of 0.84, indicating very good internal consistency. Data from the pilot study were excluded from the final analysis.

Measures

The response variable of this study was barriers to psychiatric care. BACE v3 is a comprehensive tool used to evaluate these barriers [16]. Participants' responses are measured on a scale from 0 (not at all) to 3 (a lot), with the total score ranging up to 90. Higher scores indicate more significant barriers to accessing care.

Ethical approval and informed consent

The research followed the guidelines of the Institutional Research Ethics Board and the Declaration of Helsinki. The Ethical Review Committee of the College of Health Sciences at Hawler Medical University approved the study (ethical code: Sc.E.C.6D) on April 2, 2024. All participants provided electronic informed consent before starting the survey, and they were informed about the study's aims and benefits on the first page of the survey. The privacy and confidentiality of their information were assured.

Statistical analysis

Categorical data were described using frequencies and percentages, while continuous variables were characterized by means and standard deviations. Chi-square tests, including Fisher's exact test and odds ratios (OR), were applied to the categorical data. Statistical analyses were performed using SPSS version 27 (IBM Corp., Armonk, NY), with a significance threshold set at p < 0.05.

## Results

Demographic characteristics

The study assessed 407 young adults in Erbil, providing valuable insights into various demographic variables. Females comprised a larger proportion of the sample, with 273 participants (67.1%) being female and 134 participants (32.9%) being male. The average age of the participants was 22.03 ± 1.91 years, ranging from 18 to 25 years. Most participants were single, with 59.7% of males and 74.7% of females reporting this status. The majority identified as Muslim, with 84.3% of males and 96.0% of females, while smaller percentages identified as Christian, Atheist, or with other affiliations. In terms of mental health, 6.0% of males and 2.6% of females reported currently having a mental health problem (OR = 2.71, 95% CI = 1.22 to 4.93, P = 0.043), and 14.9% of males and 11.7% of females reported having experienced a mental health issue at some point in their lives (OR = 2.11, 95% CI = 1.07 to 3.44, P = 0.005). This demographic information is detailed in Table 1.

**Table 1:** Demographic characteristics of young adults in Erbil, Iraq. Note: N = 407 represents the total number of participants in the study.

Demographic variables	Male, % (n)	Female, % (n)
Gender	32.9 (134)	67.1 (273)
Age, years		
18-19	14.9 (20)	17.2 (47)
20-21	17.9 (24)	7.0 (19)
22-23	40.3 (54)	59.3 (162)
24-25	26.9 (36)	6.5 (45)
Mean (SD)	22.03 (1.91), range = 18-25	
Relationship status		
Single	59.7 (80)	74.7 (204)
Others	40.3 (54)	25.3 (69)
Religion		
Muslim	84.3 (113)	96.0 (262)
Christian	6.0 (8)	1.8 (5)
Atheist	1.5 (2)	1.5 (4)
Others	8.2 (11)	0.7 (2)
Self-reported current mental health problem		
Yes	6.0 (8)	2.6 (7)
No	94.0 (126)	97.4 (266)
OR (95% CI), P-value	2.71 (1.22 to 4.93), P = 0.043	
Self-reported lifetime mental health problem		
Yes	14.9 (20)	11.7 (32)
No	85.1 (114)	88.3 (241
OR (95% CI), P-value	2.11 (1.07 to 3.44), P = 0.005	

Stigma-related barriers

The data show that stigma-related barriers play a significant role in accessing mental health care. For instance, the fear of being seen as weak because of mental health challenges had an average score of 2.14 ± 0.96, indicating a high level of concern with some variation among participants. Similarly, feelings of embarrassment or potential negative reactions from family members were also prominent barriers, with mean scores of 1.88 ± 1.09 and 1.75 ± 1.08, respectively. In social and work environments, fears of being labeled as "crazy" and worries about others finding out about one's mental health problems had average scores of 1.80 ± 1.19 and 1.80 ± 1.09, respectively. Apprehensions about mental illness impacting future job prospects had a mean of 1.39 ± 1.08. Concerns about appearing as an incapable parent and difficulties in managing childcare while seeking help showed lower averages of 1.24 ± 1.03 and 1.35 ± 0.98, respectively. The fear of children being placed in care due to parental mental illness had the lowest mean of 1.08 ± 0.94. These findings are further detailed in Table 2.

**Table 2:** Assessment of stigma-related barriers to psychiatric care among young adults in Erbil, Iraq. Note: N = 407 represents the total number of participants in the study.

Stigma-related barrier items	Reporting item as a barrier to any degree, % (n)	Reporting item as a major barrier, % (n)	Total (N)	Mean (SD)
Concern that I might be seen as weak for having a mental health problem	93.2 (379)	47.2 (192)	407	2.14 (0.96)
Concern that it might harm my chances when applying for jobs	73.9 (301)	20.1 (82)	407	1.39 (1.08)
Concern about what my family might think, say, do, or feel	82.3 (335)	31.2 (127)	407	1.75 (1.08)
Feeling embarrassed or ashamed	85.8 (349)	39.6 (161)	407	1.88 (1.09)
Concern that I might be seen as "crazy"	79.6 (324)	42.5 (173)	407	1.80 (1.19)
Concern that I might be seen as a bad parent	68.3 (278)	12 (49)	407	1.24 (1.03)
Concern that people I know might find out	82.8 (337)	34.4 (140)	407	1.80 (1.09)
Concern that people might not take me seriously if they found out I was having professional care	79.3 (323)	15.7 (64)	407	1.42 (0.99)
Concern that my children may be taken into care or that I may lose access or custody without my agreement	69.3 (282)	8.4 (34)	407	1.07 (0.92)
Concern about what my friends might think, say, or do	77.2 (314)	21.4 (87)	407	1.49 (1.07)
Concern about what people at work might think, say, or do	74.5 (303)	16.5 (67)	407	1.40 (1.04)
Having problems with childcare while I receive professional care	76.7 (312)	12.8 (52)	407	1.35 (0.98)

Attitudinal barriers

The results revealed various attitudinal barriers to mental health care. Handling issues independently had a high tendency, with a mean score of 2.04 ± 0.98, while the belief that problems will resolve on their own had a mean of 1.88 ± 0.98. There was substantial hesitancy toward professional intervention, as reflected by a mean score of 1.70 ± 1.08 for fears of involuntary hospitalization. Additional obstacles included a preference for alternative treatments (mean = 1.13 ± 1.11), doubts about the usefulness of conservative care (mean = 0.95 ± 0.98), and a reluctance to address personal issues (mean = 1.49 ± 1.00). Concerns about medication reactions (mean = 1.70 ± 1.06), a desire to avoid having mental health issues recorded in medical history (mean = 1.52 ± 1.14), and past negative healthcare experiences (mean = 0.84 ± 1.03) were also noted. Some participants preferred confiding in family or friends (mean = 1.17 ± 0.89) or denied having any problems (mean = 1.10 ± 0.99). For more details, please refer to Table 3.

**Table 3:** Evaluation of attitudinal barriers to psychiatric care among young adults in Erbil, Iraq. Note: N = 407 represents the total number of participants in the study.

Attitude barrier items	Reporting item as a barrier to any degree, % (n)	Reporting item as a major barrier, % (n)	Total (N)	Mean (SD)
Wanting to solve the problem on my own	91.1 (371)	41.0 (167)	407	2.04 (0.98)
Fear of being put in hospital against my will	82.6 (335)	30.5 (124)	407	1.70 (1.08)
Thinking the problem would get better by itself	90.2 (367)	32.7 (133)	407	1.88 (0.98)
Preferring to get alternative forms of care (e.g. traditional/religious healing or alternative/complementary therapies)	61.4 (250)	16.7 (68)	407	1.13 (1.11)
Thinking that professional care probably would not help	57.3 (233)	7.9 (32)	407	0.95 (0.98)
Dislike talking about my feelings, emotions, or thoughts	80.1 (326)	17.0 (69)	407	1.49 (1.0)
Concerns about the treatments available (e.g. medication side effects)	84.3 (343)	29.5 (120)	407	1.70 (1.06)
Not wanting a mental health problem to be on my medical records	75.7 (308)	27.5 (112)	407	1.52 (1.14)
Having had previous bad experiences with professional care for mental health	47.4 (193)	9.3 (38)	407	0.84 (1.03)
Preferring to get help from family or friends	74.2 (302)	6.1 (25)	407	1.17 (0.89)
Thinking I did not have a problem	69.0 (281)	14.0 (57)	407	1.10 (0.99)

Instrumental barriers

The data also revealed several instrumental obstacles to accessing mental health support. One significant barrier was the limited availability of mental health experts from one's own cultural or ethnic group, with an average of 1.78 ± 1.09, highlighting the importance of provider diversity. Additionally, there was a prevalent lack of knowledge about where to find professional assistance, with a mean score of 1.16 ± 1.04. Practical challenges included transportation problems (mean = 0.61 ± 0.87), unaffordable care costs (mean = 0.92 ± 1.01), and severe illness that prevented help-seeking (mean = 0.85 ± 0.95). Difficulties in obtaining work leave for appointments (mean = 1.16 ± 1.11) and a lack of assistance in seeking treatment (mean = 1.03 ± 0.98) were also significant logistical barriers. Further details can be found in Table 4.

**Table 4:** Analysis of instrumental barriers to accessing psychiatric care among young adults in Erbil, Iraq. N = 407 represents the total number of participants in the study.

Instrumental barrier items	Reporting item as a barrier to any degree, % (n)	Reporting item as a major barrier, % (n)	Total (N)	Mean (SD)
Being unsure where to go to get professional care	69.3 (282)	16.7 (68)	407	1.16 (1.04)
Problems with transport or traveling to appointments	40.8 (164)	5.7 (23)	407	0.61 (0.87)
Not being able to afford the financial costs involved	54.6 (222)	9.6 (39)	407	0.92 (1.01)
Professionals from my own ethnic or cultural group not being available	83.5 (340)	34.2 (139)	407	1.78 (1.09)
Being too unwell to ask for help	56.0 (227)	9.8 (40)	407	0.85 (0.95)
Difficulty taking time off work	62.9 (256)	17.2 (70)	407	1.16 (1.11)
Having no one who could help me get professional care	64.1 (261)	10.1 (41)	407	1.03 (0.98)

## Discussion

The present study aimed to investigate the reasons why young adults in Erbil, Iraq are hesitant to seek help from psychiatric hospitals and to identify the barriers to receiving psychiatric care. The findings provide insight into the different factors that contribute to this reluctance, such as stigma, attitudes, and practical obstacles. In summary, the results reveal a worrisome level of hesitancy and avoidance when it comes to seeking professional mental health support among young adults in this region.

Mental health disorders are a major global public health concern, causing a significant burden of disease and disability [18]. Young adults are especially vulnerable, as many mental health conditions arise during this developmental stage [7]. It is crucial to address mental health challenges early on to prevent long-term negative consequences and promote overall well-being. However, barriers to accessing mental health services can impede timely intervention and exacerbate the impact of these conditions. Recognizing the critical need to understand these barriers, the researchers endeavored to assess young adults' levels of barriers to psychiatric care in the Kurdish society of Erbil, Iraq.

The demographic analysis revealed that the majority of participants were females, which is consistent with other studies indicating that women are more likely to participate in mental health research and report mental health issues [19,20]. The average age of the participants, 22.03 years, falls within the typical range for young adults, who are particularly vulnerable to developing mental health conditions such as depression and anxiety during this developmental stage [21]. Most participants were single, which aligns with the demographic characteristics of young adult populations [22]. The high percentage of Muslim participants reflects the predominant religion in Erbil, providing a culturally relevant context for understanding mental health barriers.

In terms of mental health, a higher proportion of males reported current mental health problems compared to females, highlighting potential gender differences in mental health experiences and help-seeking behaviors [23]. The data also showed that a significant number of both males and females had experienced mental health issues at some point in their lives, emphasizing the widespread nature of mental health challenges among young adults [24]. These findings underscore the importance of addressing mental health needs in this demographic and understanding how demographic factors such as gender, age, and religion influence the barriers to accessing psychiatric care. This comprehensive demographic analysis enhances our understanding of the socio-cultural context and provides a foundation for developing targeted interventions to improve mental health access for young adults in Erbil.

Stigma-related barriers emerged as a prominent theme in the study, reflecting the pervasive societal stigma surrounding mental illness. Among participants, fears of being perceived as weak were significant, as mental health issues are often seen as a sign of personal failure rather than a legitimate health concern [25]. This perception can be attributed to cultural norms that prioritize strength and resilience, particularly in collectivistic societies where the reputation of the family and community is paramount [26]. Consequently, individuals may avoid seeking help to protect their social standing, which leads to untreated mental health conditions. Prominent feelings of embarrassment and concerns about negative reactions from family members were also observed. Apprehensions about being labeled as "crazy" and worries about others discovering their mental health problems were prevalent, highlighting the profound impact of stigma on help-seeking behaviors. Being labeled as "crazy" carries severe social penalties, including loss of status and employment opportunities. These findings align with previous research that consistently identifies stigma as a major obstacle to accessing mental health services [9,27]. Stigma leads to feelings of shame, social isolation, and discrimination, perpetuating the cycle of reluctance to seek help and exacerbating the negative consequences of untreated mental health conditions [28,29].

Attitudinal barriers played a significant role in deterring young adults from engaging with psychiatric hospitals. Prominent attitudes included a tendency to handle issues independently and a belief that problems will be resolved on their own. These attitudes reflect a preference for self-reliance and a potential underestimation of the severity or persistence of mental health challenges [30]. Cultural values that emphasize personal strength and resilience may influence this self-reliance, leading individuals to believe they should manage their problems without external help. Additionally, fears of involuntary hospitalization and a reluctance to address personal issues contribute to hesitancy toward professional intervention [31]. These concerns can stem from negative perceptions of psychiatric care and a lack of trust in mental health professionals. Dramatized media portrayals of psychiatric institutions often exacerbate these negative perceptions [32]. In the context of Erbil, beliefs about the causes of mental illness, such as attributing symptoms to supernatural factors or moral failings, can further intensify stigma and discourage individuals from seeking professional help. These cultural beliefs may lead to the perception that mental health issues should be dealt with privately or through traditional healing practices rather than formal psychiatric care. This underscores the importance of culturally tailored educational initiatives to address these specific misconceptions and promote a better understanding of mental health.

Instrumental barriers further compounded the challenges in accessing psychiatric care. The limited availability of mental health experts from the participants' own cultural or ethnic backgrounds was a significant concern, underscoring the importance of provider diversity and cultural competence in mental health service delivery [33]. Additionally, a lack of knowledge about where to find professional assistance emerged as a barrier, suggesting a need for better dissemination of information about available resources. Practical challenges, such as transportation problems, unaffordable care costs, and difficulties in obtaining work leave for appointments, also hindered access to mental health services [34]. In the context of Erbil, decisions to seek help and manage finances for young adults are often influenced by family members, particularly parents, due to the collectivistic nature of society. This familial influence can either facilitate or impede access to mental health services, depending on the family's attitudes toward mental health. These findings align with previous research highlighting the impact of logistical and financial barriers on help-seeking behaviors, particularly in low- and middle-income countries [35,36]. Addressing these instrumental barriers by improving healthcare infrastructure, providing financial support, and increasing public awareness about available mental health services can significantly enhance access and engagement in mental health care [37-39].

The study's findings have important implications for addressing the mental health needs of young adults in Erbil and other similar contexts. Comprehensive strategies are needed to overcome the multifaceted barriers identified, including efforts to reduce stigma, increase mental health literacy, improve access to culturally competent care, and address practical obstacles [40]. Additionally, community-based interventions and collaborations with local organizations could help promote mental health awareness, facilitate early identification of mental health concerns, and provide support services tailored to the specific needs and cultural context of young adults in Erbil. However, a notable limitation of this study is its primary focus on Erbil, which may limit the generalizability of its findings to other regions. Furthermore, the higher number of female participants compared to males may introduce a gender bias, affecting the applicability of the findings across genders. Future research should focus on evaluating the effectiveness of these interventions and identifying additional culturally specific barriers to mental health care.

## Conclusions

The results of this study revealed significant stigma-related, attitudinal, and instrumental barriers to mental health care among young adults in Erbil. It highlighted that young adults, in particular, are facing diverse barriers significantly influenced by cultural factors. In response to these findings, it is recommended for governments, especially in regions like Erbil, to prioritize enhancing mental health awareness, actively work toward destigmatizing mental health issues, and improve the accessibility and suitability of mental health services. Implementing mental health education within school curricula and launching comprehensive public awareness campaigns are key steps. These actions, along with ensuring the availability of culturally sensitive and easily accessible mental health resources, are essential for fostering a more inclusive and supportive environment for mental health care

## Appendices

Questionnaire

Our questionnaire is divided into two main parts: sociodemographic characteristics of the patient and general questions about barriers to access to care evaluation. To investigate the factors that influence the reluctance of young adults in Erbil, Iraq to seek psychiatric care and engage with psychiatric hospitals.

Code .....…

Part 1: Sociodemographic characteristics

Section one: Sociodemographic characteristics of students

X1: Age: ………..

X2: Gender:

1. Male

2. Female

X3: Relationship status:

1. Single

2. Others

X4: Religion:

1. Muslim

2. Christian

3. No religion

4. Others

X5. Self-reported current mental health problem:

1. Yes

2. No

X6. Self-reported lifetime mental health problem:

1. Yes

2. No

Part 2: Barriers to Access to Care Evaluation (BACE v3)1

Below you can see a list of things that can stop, delay, or discourage people from getting professional care for a mental health problem or continuing to get help. By professional care, we mean care from such staff as a general practitioner (family doctor), community mental health team (e.g. care coordinator, mental health nurse, or mental health social worker), psychiatrist, counselor, psychologist, or psychotherapist.

Have any of these issues ever stopped, delayed, or discouraged you from getting, or continuing with, professional care for a mental health problem?

Please circle one number on each row to indicate the answer that best suits you.

For "not applicable", e.g. if it is a question about children and you do not have children, please cross the "Not applicable" box.

**Table 5:** Barriers to Access to Care Evaluation (BACE v3)1 Note: © 2024 King's College London. All rights reserved. Permission is granted for use by researchers provided no changes are made to the wording or format of the BACE. For more information, contact Professor Graham Thornicroft (graham.thornicroft@iop.kcl.ac.uk) or Dr. Sarah Clement (sarah.clement@kcl.ac.uk).

	Issue	This has stopped, delayed, or discouraged me "NOT AT ALL"	This has stopped, delayed, or discouraged me "A LITTLE"	This has stopped, delayed, or discouraged me "QUITE A LOT"	This has stopped, delayed, or discouraged me "A LOT"
1.	Being unsure where to go to get professional care	0	1	2	3
2.	Wanting to solve the problem on my own	0	1	2	3
3.	Concern that I might be seen as weak for having a mental health problem	0	1	2	3
4.	Fear of being put in hospital against my will	0	1	2	3
5.	Concern that it might harm my chances when applying for jobs. Not applicable □	0	1	2	3
6.	Problems with transport or traveling to appointments	0	1	2	3
7.	Thinking the problem would get better by itself	0	1	2	3
8.	Concern about what my family might think, say, do, or feel	0	1	2	3
9.	Feeling embarrassed or ashamed	0	1	2	3
10.	Preferring to get alternative forms of care (e.g. traditional/religious healing or alternative/complementary therapies)	0	1	2	3
11.	Not being able to afford the financial costs involved	0	1	2	3
12.	Concern that I might be seen as "crazy"	0	1	2	3
13.	Thinking that professional care probably would not help	0	1	2	3
14.	Concern that I might be seen as a bad parent. Not applicable □	0	1	2	3
15.	Professionals from my own ethnic or cultural group not being available	0	1	2	3
16.	Being too unwell to ask for help	0	1	2	3
17.	Concern that people I know might find out	0	1	2	3
18.	Dislike talking about my feelings, emotions, or thoughts	0	1	2	3
19.	Concern that people might not take me seriously if they found out I was having professional care	0	1	2	3
20.	Concerns about the treatments available (e.g. medication side effects)	0	1	2	3
21	Not wanting a mental health problem to be on my medical records	0	1	2	3
22.	Having had previous bad experiences with professional care for mental health	0	1	2	3
23.	Preferring to get help from family or friends	0	1	2	3
24.	Concern that my children may be taken into care or that I may lose access or custody without my agreement. Not applicable □	0	1	2	3
25.	Thinking I did not have a problem	0	1	2	3
26.	Concern about what my friends might think, say, or do	0	1	2	3
27.	Difficulty taking time off work. Not applicable □	0	1	2	3
28.	Concern about what people at work might think, say, or do. Not applicable □	0	1	2	3
29.	Having problems with childcare while I receive professional care. Not applicable □	0	1	2	3
30.	Having no one who could help me get professional care	0	1	2	3
